# Synthesis and structure of photodegradable 1-(4,5-dimeth­oxy-2,3-di­nitro­phen­yl)-2-methyl­propyl *N*-butyl­carbamate

**DOI:** 10.1107/S2056989023004103

**Published:** 2023-05-16

**Authors:** Takafumi Honda, Michiko Ito, Kazuo Yamaguchi, Noriko Chikaraishi Kasuga, Hiroyasu Sato

**Affiliations:** a Department of Chemistry, Faculty of Science, Kanagawa University, Tsuchiya, Hiratsuka, Kanagawa 259-1293, Japan; b Rigaku Corporation 3-9-12 Matsubara-cho, Akishima, Tokyo 196-8666, Japan; Universidade de Sâo Paulo, Brazil

**Keywords:** photolabile carbamate, 2,3-di­nitro­benzyl group, inter­molecular hydrogen bonding, crystal structure

## Abstract

The reaction of 1-(4,5-dimeth­oxy-2,3-di­nitro­phen­yl)-2-methyl­propan-1-ol and butyl­iso­cyanate using di­butyl­tin dilaurate as a catalyst afforded 1-(4,5-dimeth­oxy-2,3-di­nitro­phen­yl)-2-methyl­propyl *N*-butyl­carbamate, C_17_H_25_N_3_O_8_, which released butyl­amine upon photoirradiation. Single crystals of the title compound were grown in a 1:1 mixed solution of hexane and ethyl acetate. Two nitro groups and one meth­oxy group are twisted out of the plane of the aromatic ring in the novel photo-protecting group. Inter­molecular hydrogen bonds are observed between *N*-butyl­carbamate moieties parallel to the *a* axis.

## Abstract 2

1-(4,5-Dimeth­oxy-2,3-di­nitro­phen­yl)-2-methyl­propyl butyl­carbamate, which affords butyl­amine on photoirradiation, was prepared by the reaction of 1-(4,5-dimeth­oxy-2,3-di­nitro­phen­yl)-2-methyl­propan-1-ol and butyl­iso­cyanate using di­butyl­tin dilaurate as a catalyst. Single crystals of the carbamate were grown in a 1:1 mixed solution of hexane and ethyl acetate. Two nitro groups and one meth­oxy group of the novel photo-protecting group were twisted out of the plane of the aromatic ring and inter­molecular hydrogen bonds are observed between carbamate moieties.

## 1. Chemical context

Photodegradable protective groups (PPGs) are common tools in the field of organic, biochemical, and materials sciences (Cambie *et al.*, 2016 ▸; Ellis-Davies, 2020 ▸; Hansen *et al.*, 2015 ▸). 2-Nitro­benzyl groups are widely used as PPGs because several precursors are available, they react with various functional groups and the resulting protected compounds are soluble in common organic solvents. To increase the sensitivity of 2-nitro­benzyl-group-protected derivatives, several chemical modifications, such as the introduction of substituents at the α-position, have been performed (Kasuga *et al.*, 2016 ▸; Zhao *et al.*, 2012 ▸). 
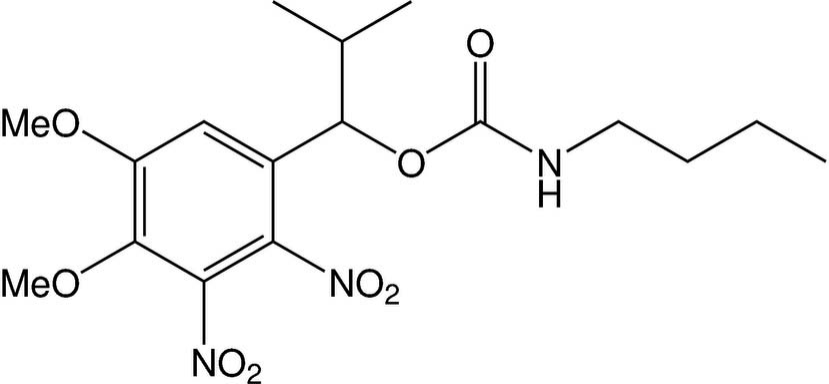


Herein, we report the synthesis and structure of the novel photolabile title compound 1-(4,5-dimeth­oxy-2,3-di­nitro­phen­yl)-2-methyl­propyl *N*-butyl­carbamate (**1**), which has an isopropyl group at the α-position and an additional nitro group at the 3-position. It was prepared from the corres­ponding alcohol and iso­cyanate using a tin catalyst based on a previously reported reaction with modifications (Stegmaier *et al.*, 2008 ▸) and the amine was released upon photoirradiation, which indicated that the compound has high reactivity and the potential for application in various functional materials. Compound **1** exhibits a higher sensitivity to light than the corresponding compound with one nitro group at the *ortho* position (Kasuga *et al.*, 2015 ▸).

## 2. Structural commentary

**Figure 1:**
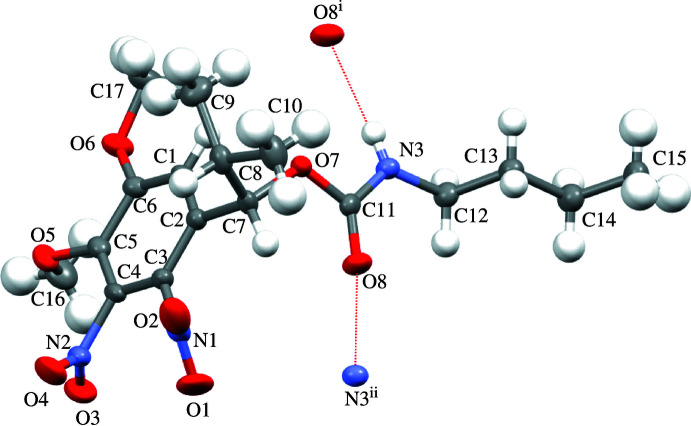
Mol­ecular structure of photocleavable 1-(4,5-dimeth­oxy-2,3-di­nitro­phen­yl)-2-methyl­propyl butyl­carbamate. Displacement ellipsoids are drawn at the 50% probability level. The inter­molecular hydrogen bonds (Table 1 ▸) are drawn as dotted lines.

The asymmetric unit of **1** contains a single mol­ecule in which four functional groups and a carbamate linkage site are connected to the aromatic ring. The substituents are positioned in a manner that prevents steric repulsion (Fig. 1 ▸).

The aromatic ring (C1–C6) and five attached atoms (N1, N2, O5, O6, and C7) are almost planar, as indicated by the torsion angles N2—C3—C2—N1 = 4.4 (3)°, O5—C4—C5—O6 = −8.0 (3)°, and C7—C1—C2—N1 = −4.3 (3)°. However, the torsion angles involving the two nitro groups [O2—N1—C2—C1 = 56.9 (3)°, O1—N1—C2—C1 = −122.5 (2)°, O3—N2—C3—C2 = −125.9 (3)°, and O4—N2—C3—C2 = 53.9 (3)°] indicate that both NO_2_ groups are twisted relative to the plane of the aromatic ring. The torsion angles involving the two meth­oxy groups [C16—O5—C4—C3 and C17—O6—C5—C4) are −110.7 (2)° and 172.5 (2)°] indicate that one meth­oxy group (O5–C16) is twisted and the other (O6–C17) is located in the same plane as the aromatic ring.

In the related compound, 4,5-dimeth­oxy-2-nitro­benzyl acetate (shown in the scheme below[Chem scheme2]), the nitro and dimeth­oxy groups are in the same plane as the aromatic ring and the whole mol­ecule is flat in the absence of substituents at the α-position (Kasuga *et al.*, 2015 ▸). 
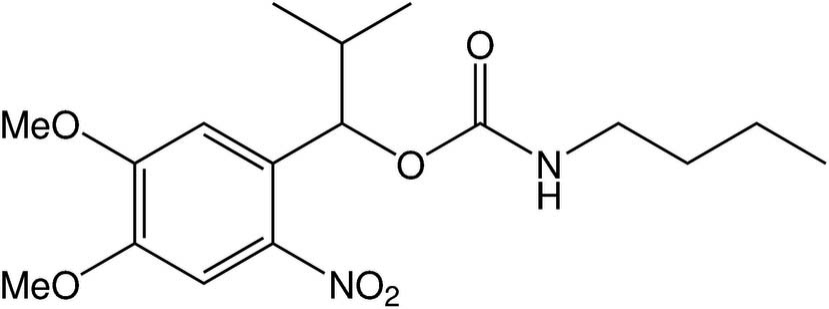


## 3. Supra­molecular features

**Figure 2:**
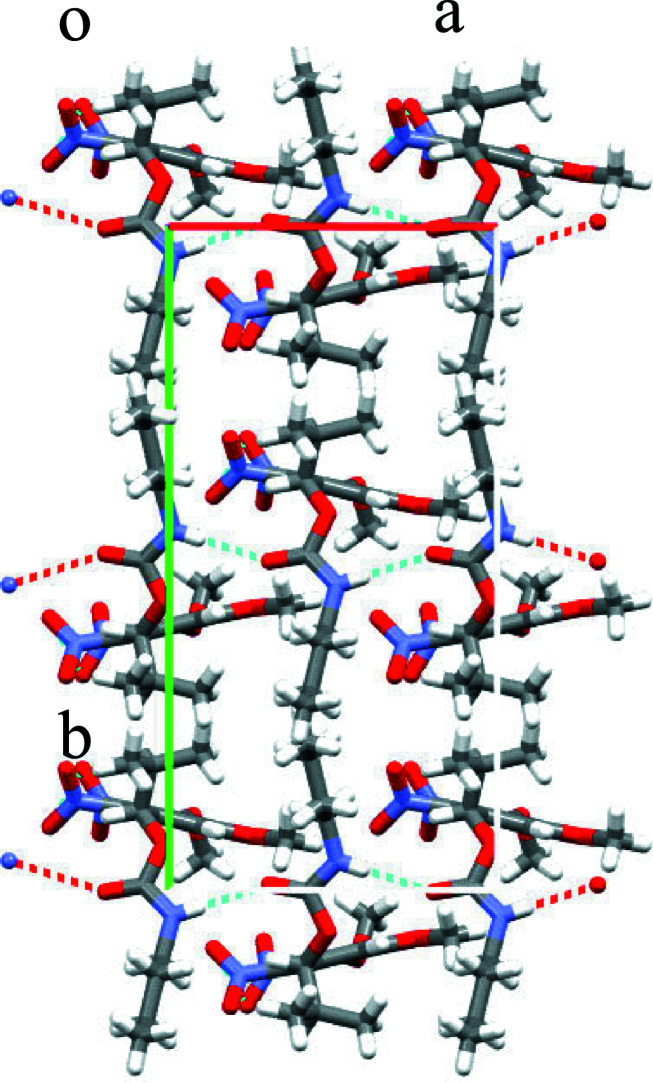
Inter­molecular hydrogen bonds (Table 1 ▸) between the carbamate moieties, shown as dotted lines, link the molecules into chains parallel to the *a* axis.

In the crystal, N3—H3⋯O8^i^ hydrogen bonds link the mol­ecules into chains parallel to the *a* axis (Fig. 2 ▸ and Table 1 ▸).

## 4. Synthesis and crystallization

The synthesis of compound **1** was based on the method reported by Stegmaier *et al.* (2008 ▸) with modifications. The synthesis and photocleavage is shown in the scheme below[Chem scheme3]. This di­nitro­benzyl protecting group photocleaved twice as fast as the corresponding 2-mono­nitro­benzyl group. Under an N_2_ atmosphere, a mixture of 1-(4,5-dimeth­oxy-2,3-di­nitro­phen­yl)-2-methyl­propan-1-ol (150 mg, 0.5 mmol), *n*-butyl iso­cyanate (85 µ*L*, 0.75 mmol), di­butyl­tin laurate (15 µ*L*), and 1.5 ml of tetra­hydro­furan were refluxed for 19 h. After removing the solvent, the crude solid was purified by silica gel column chromatography. Pale-yellow needle-shaped crystals were grown in a 1:1 mixed solution of hexane and ethyl acetate by slow evaporation. The crystals were washed with a small volume of ethyl acetate and dried *in vacuo* (50 mg). 
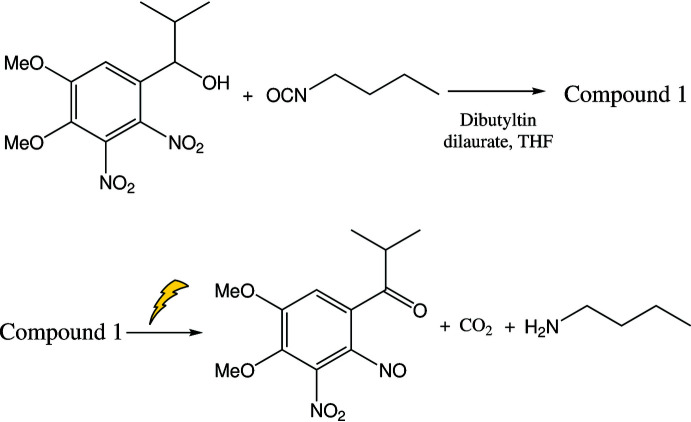


Analysis calculated for C_17_H_25_N_3_O_8_: C 51.12, H 6.31, N 10.52%. Found: C 51.07, H 6.20, N 10.42%. Prominent IR bands at 1800–400 cm^−1^ (KBr disc): 1693*m*, 1557*m*, 1359*m*. Melting point 379.9–381.1 K (uncorrected). ^1^H NMR (CDCl_3_, 292.2 K) 0.90 (3H, *t*), 0.91 (3H, *d*), 1.03 (3H, *d*), 1.32 (2H, *m*), 1.45 (2H, *m*), 2.18 (1H, *m*), 3.13 (2H, *m*), 3.93 (6H, *s*), 4.76 (1H, *br*), 5.61 (1H, *d*), 6.97 (1H, *s*). ^13^C NMR (CDCl_3_, 293.7 K) 13.69, 17.88, 19.21, 19.86, 31.88, 33.39, 40.85, 56.71, 62.58, 76.04, 111.29, 131.17, 134.82, 140.45, 141.14, 155.28, 155.84.

## 5. Refinement

Crystal data, data collection and structure refinement details are summarized in Table 2 ▸. The C-bound H atoms were positioned geometrically (C—H = 0.93–0.98 Å) and refined using a riding model with *U*_iso_(H) = 1.2 or 1.5*U*_eq_(C).

## Supplementary Material

Crystal structure: contains datablock(s) I, global. DOI: 10.1107/S2056989023004103/ex2069sup1.cif

Structure factors: contains datablock(s) I. DOI: 10.1107/S2056989023004103/ex2069Isup2.hkl

Supporting information file. DOI: 10.1107/S2056989023004103/ex2069Isup4.cdx

Supporting information file. DOI: 10.1107/S2056989023004103/ex2069Isup4.cml

CCDC reference: 2261578

Additional supporting information:  crystallographic information; 3D view; checkCIF report

## Figures and Tables

**Table 1:** Hydrogen-bond geometry (Å, °)

*D*—H⋯*A*	*D*—H	H⋯*A*	*D*⋯*A*	*D*—H⋯*A*
N3—H3⋯O8^i^	0.78 (3)	2.26 (3)	3.042 (3)	176 (3)

Symmetry code: (i) 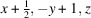 .

**Table 2:** Experimental details

Crystal data	
Chemical formula	C_17_H_25_N_3_O_8_
*M*_r_	399.40
Crystal system, space group	Monoclinic, *I* *a*
Temperature (K)	120
*a*, *b*, *c* (Å)	9.9490 (4), 19.9223 (8), 10.2378 (4)
β (°)	97.616 (4)
*V* (Å^3^)	2011.30 (14)
*Z*	4
Radiation type	Mo *K*α
μ (mm^−1^)	0.11
Crystal size (mm)	0.25 × 0.21 × 0.18

Data collection	
Diffractometer	Rigaku VariMax SaturnCCD724
Absorption correction	Multi-scan (*CrysAlis PRO*; Rigaku OD, 2018 ▸)
*T*_min_, *T*_max_	0.906, 1.000
No. of measured, independent and observed [*I* > 2σ(*I*)] reflections	18806, 5927, 4720
*R*_int_	0.039
(sin θ/λ)_max_ (Å^−1^)	0.730

Refinement	
*R*[*F* ^2^ > 2σ(*F* ^2^)], *wR*(*F* ^2^), *S*	0.045, 0.094, 1.04
No. of reflections	5927
No. of parameters	262
No. of restraints	2
H-atom treatment	H atoms treated by a mixture of independent and constrained refinement
Δρ_max_, Δρ_min_ (e Å^−3^)	0.18, −0.19

Computer programs: *CrysAlis PRO* (Rigaku OD, 2018 ▸), *SHELXT* (Sheldrick, 2015*a* ▸), *SHELXL* (Sheldrick, 2015*b* ▸), and *OLEX2* (Dolomanov *et al.*, 2009 ▸).

## supplementary crystallographic information

### Crystal data

**Table:**

C_17_H_25_N_3_O_8_	*D*_x_ = 1.319 Mg m^−^^3^
*M**_r_* = 399.40	Melting point = 380.1–274.2 K
Monoclinic, *I**a*	Mo *K*α radiation, λ = 0.71073 Å
*a* = 9.9490 (4) Å	Cell parameters from 10281 reflections
*b* = 19.9223 (8) Å	θ = 3.4–31.2°
*c* = 10.2378 (4) Å	µ = 0.11 mm^−^^1^
β = 97.616 (4)°	*T* = 120 K
*V* = 2011.30 (14) Å^3^	Block, clear light yellow
*Z* = 4	0.25 × 0.21 × 0.18 mm
*F*(000) = 848	

### Data collection

**Table:**

Rigaku VariMax SaturnCCD724 diffractometer	5927 independent reflections
Radiation source: fine-focus sealed X-ray tube, Enhance (Mo) X-ray Source	4720 reflections with *I* > 2σ(*I*)
Graphite monochromator	*R*_int_ = 0.039
ω scans	θ_max_ = 31.3°, θ_min_ = 3.4°
Absorption correction: multi-scan (CrysAlisPro; Rigaku OD, 2018)	*h* = −14→14
*T*_min_ = 0.906, *T*_max_ = 1.000	*k* = −27→28
18806 measured reflections	*l* = −14→14

### Refinement

**Table:**

Refinement on *F*^2^	Primary atom site location: dual
Least-squares matrix: full	Hydrogen site location: mixed
*R*[*F*^2^ > 2σ(*F*^2^)] = 0.045	H atoms treated by a mixture of independent and constrained refinement
*wR*(*F*^2^) = 0.094	*w* = 1/[σ^2^(*F*_o_^2^) + (0.040*P*)^2^ + 0.1525*P*] where *P* = (*F*_o_^2^ + 2*F*_c_^2^)/3
*S* = 1.03	(Δ/σ)_max_ < 0.001
5927 reflections	Δρ_max_ = 0.18 e Å^−^^3^
262 parameters	Δρ_min_ = −0.18 e Å^−^^3^
2 restraints	

### Special details

**Table:**

Geometry. All esds (except the esd in the dihedral angle between two l.s. planes) are estimated using the full covariance matrix. The cell esds are taken into account individually in the estimation of esds in distances, angles and torsion angles; correlations between esds in cell parameters are only used when they are defined by crystal symmetry. An approximate (isotropic) treatment of cell esds is used for estimating esds involving l.s. planes.

### Fractional atomic coordinates and isotropic or equivalent isotropic displacement parameters (Å^2^)

**Table:**

	*x*	*y*	*z*	*U*_iso_*/*U*_eq_	
O1	0.13662 (19)	0.40830 (14)	0.6373 (2)	0.0649 (7)	
O2	0.18717 (19)	0.32139 (11)	0.5256 (2)	0.0512 (5)	
O3	0.2890 (2)	0.41867 (10)	0.9547 (2)	0.0502 (5)	
O4	0.2377 (2)	0.32211 (10)	0.8644 (2)	0.0483 (5)	
O5	0.57340 (17)	0.39172 (8)	0.96576 (16)	0.0313 (4)	
O6	0.75485 (16)	0.42284 (9)	0.80062 (16)	0.0341 (4)	
O7	0.48515 (15)	0.43395 (7)	0.34698 (15)	0.0246 (3)	
O8	0.30610 (16)	0.50552 (8)	0.34710 (17)	0.0349 (4)	
N1	0.2168 (2)	0.36864 (14)	0.5995 (2)	0.0403 (6)	
N2	0.3014 (2)	0.37462 (11)	0.8748 (2)	0.0346 (5)	
N3	0.5126 (2)	0.54139 (9)	0.30272 (19)	0.0248 (4)	
C1	0.4522 (2)	0.38928 (11)	0.5581 (2)	0.0242 (5)	
C2	0.3608 (2)	0.38015 (11)	0.6472 (2)	0.0266 (5)	
C3	0.4010 (2)	0.38545 (11)	0.7824 (2)	0.0270 (5)	
C4	0.5334 (2)	0.39790 (11)	0.8338 (2)	0.0260 (5)	
C5	0.6271 (2)	0.40978 (11)	0.7437 (2)	0.0255 (5)	
C6	0.5857 (2)	0.40607 (11)	0.6096 (2)	0.0249 (5)	
C7	0.4121 (2)	0.38328 (11)	0.4104 (2)	0.0262 (5)	
C8	0.4431 (3)	0.31488 (12)	0.3535 (2)	0.0309 (5)	
C9	0.5937 (3)	0.29733 (13)	0.3712 (3)	0.0413 (6)	
C10	0.3836 (3)	0.31118 (14)	0.2093 (3)	0.0426 (7)	
C11	0.4244 (2)	0.49560 (11)	0.3336 (2)	0.0233 (4)	
C12	0.4751 (2)	0.61096 (11)	0.2740 (2)	0.0288 (5)	
C13	0.4652 (2)	0.62909 (11)	0.1295 (2)	0.0289 (5)	
C14	0.4296 (3)	0.70286 (12)	0.1055 (3)	0.0374 (6)	
C15	0.4166 (3)	0.72204 (14)	−0.0381 (3)	0.0421 (7)	
C16	0.6073 (3)	0.45403 (14)	1.0351 (3)	0.0446 (7)	
C17	0.8593 (2)	0.42724 (15)	0.7164 (3)	0.0412 (7)	
H3	0.588 (3)	0.5291 (12)	0.310 (3)	0.026 (7)*	
H6	0.648082	0.414974	0.551752	0.030*	
H7	0.314682	0.392050	0.389922	0.031*	
H8	0.397129	0.280852	0.400680	0.037*	
H9A	0.642527	0.331190	0.330187	0.062*	
H9B	0.606179	0.254638	0.331034	0.062*	
H9C	0.627043	0.295128	0.463472	0.062*	
H10A	0.288238	0.320714	0.200686	0.064*	
H10B	0.397234	0.266968	0.176167	0.064*	
H10C	0.427650	0.343506	0.159920	0.064*	
H12A	0.541687	0.639913	0.323654	0.035*	
H12B	0.388247	0.619657	0.303969	0.035*	
H13A	0.396452	0.601434	0.079559	0.035*	
H13B	0.551210	0.619674	0.098363	0.035*	
H14A	0.344649	0.712310	0.138677	0.045*	
H14B	0.499305	0.730341	0.154638	0.045*	
H15A	0.500225	0.712690	−0.071672	0.063*	
H15B	0.396447	0.769065	−0.047350	0.063*	
H15C	0.344706	0.696579	−0.086689	0.063*	
H16A	0.675426	0.477282	0.994558	0.067*	
H16B	0.641171	0.444566	1.125445	0.067*	
H16C	0.527663	0.481566	1.031391	0.067*	
H17A	0.839687	0.463759	0.655666	0.062*	
H17B	0.862726	0.386057	0.668233	0.062*	
H17C	0.945213	0.434835	0.768997	0.062*	

### Atomic displacement parameters (Å^2^)

**Table:**

	*U*^11^	*U*^22^	*U*^33^	*U*^12^	*U*^13^	*U*^23^
O1	0.0257 (10)	0.1028 (19)	0.0698 (16)	0.0228 (11)	0.0194 (10)	0.0230 (14)
O2	0.0328 (10)	0.0666 (14)	0.0513 (13)	−0.0210 (10)	−0.0052 (9)	0.0176 (11)
O3	0.0577 (13)	0.0498 (12)	0.0502 (13)	0.0163 (10)	0.0334 (10)	0.0051 (9)
O4	0.0390 (11)	0.0518 (13)	0.0586 (14)	−0.0083 (9)	0.0227 (9)	0.0144 (10)
O5	0.0398 (10)	0.0322 (9)	0.0233 (8)	−0.0001 (7)	0.0097 (7)	−0.0013 (7)
O6	0.0253 (8)	0.0503 (11)	0.0274 (9)	−0.0058 (7)	0.0067 (7)	−0.0059 (7)
O7	0.0201 (7)	0.0261 (8)	0.0288 (8)	0.0013 (6)	0.0069 (6)	0.0082 (6)
O8	0.0193 (8)	0.0366 (9)	0.0498 (11)	0.0031 (7)	0.0078 (7)	0.0056 (8)
N1	0.0195 (10)	0.0607 (15)	0.0417 (13)	0.0005 (10)	0.0079 (9)	0.0249 (11)
N2	0.0297 (11)	0.0389 (12)	0.0384 (12)	0.0114 (10)	0.0170 (9)	0.0152 (10)
N3	0.0199 (9)	0.0250 (10)	0.0301 (10)	0.0015 (8)	0.0051 (8)	0.0023 (7)
C1	0.0215 (11)	0.0249 (11)	0.0269 (12)	0.0025 (8)	0.0059 (9)	0.0063 (9)
C2	0.0197 (10)	0.0296 (12)	0.0316 (13)	0.0027 (9)	0.0073 (9)	0.0087 (9)
C3	0.0256 (11)	0.0253 (11)	0.0333 (12)	0.0052 (9)	0.0157 (9)	0.0087 (9)
C4	0.0298 (12)	0.0239 (11)	0.0259 (12)	0.0032 (9)	0.0105 (9)	0.0008 (8)
C5	0.0234 (11)	0.0259 (11)	0.0280 (12)	0.0006 (9)	0.0067 (9)	−0.0016 (9)
C6	0.0208 (10)	0.0283 (12)	0.0278 (12)	−0.0005 (9)	0.0114 (9)	0.0032 (9)
C7	0.0206 (10)	0.0303 (12)	0.0278 (12)	−0.0034 (9)	0.0034 (9)	0.0104 (9)
C8	0.0343 (13)	0.0287 (12)	0.0293 (12)	−0.0065 (10)	0.0021 (10)	0.0062 (9)
C9	0.0410 (15)	0.0332 (14)	0.0485 (17)	0.0066 (12)	0.0019 (12)	−0.0069 (12)
C10	0.0525 (16)	0.0427 (15)	0.0310 (14)	−0.0119 (13)	−0.0006 (12)	0.0028 (11)
C11	0.0228 (11)	0.0278 (11)	0.0189 (10)	0.0037 (9)	0.0008 (8)	0.0024 (8)
C12	0.0302 (12)	0.0232 (11)	0.0332 (13)	0.0023 (9)	0.0050 (10)	0.0002 (9)
C13	0.0252 (11)	0.0268 (12)	0.0351 (13)	0.0001 (9)	0.0052 (9)	0.0028 (10)
C14	0.0370 (14)	0.0301 (13)	0.0459 (16)	0.0013 (11)	0.0087 (12)	0.0065 (11)
C15	0.0353 (14)	0.0404 (16)	0.0510 (17)	0.0019 (12)	0.0071 (12)	0.0169 (13)
C16	0.0603 (19)	0.0421 (16)	0.0335 (15)	−0.0066 (13)	0.0147 (13)	−0.0108 (11)
C17	0.0259 (13)	0.0651 (19)	0.0339 (15)	−0.0097 (12)	0.0085 (11)	−0.0077 (13)

### Geometric parameters (Å, º)

**Table:**

O2—N1	1.219 (3)	C8—C9	1.525 (4)
O3—N2	1.217 (3)	C8—C10	1.518 (4)
O5—C4	1.363 (3)	C9—H9A	0.9600
O5—C16	1.448 (3)	C9—H9B	0.9600
O8—C11	1.220 (3)	C9—H9C	0.9600
O6—C5	1.351 (3)	C10—H10A	0.9600
O6—C17	1.438 (3)	C10—H10B	0.9600
O7—C11	1.367 (3)	C10—H10C	0.9600
O7—C7	1.447 (2)	C12—H12A	0.9700
N1—O1	1.222 (3)	C12—H12B	0.9700
N1—C2	1.469 (3)	C12—C13	1.513 (3)
N2—O4	1.220 (3)	C13—H13A	0.9700
N2—C3	1.473 (3)	C13—H13B	0.9700
N3—C11	1.332 (3)	C13—C14	1.524 (3)
N3—C12	1.455 (3)	C14—H14A	0.9700
N3—H3	0.78 (3)	C14—H14B	0.9700
C1—C6	1.402 (3)	C14—C15	1.508 (4)
C1—C2	1.383 (3)	C15—H15A	0.9600
C1—C7	1.517 (3)	C15—H15B	0.9600
C3—C2	1.394 (3)	C15—H15C	0.9600
C4—C3	1.374 (3)	C16—H16A	0.9600
C4—C5	1.416 (3)	C16—H16B	0.9600
C6—H6	0.9300	C16—H16C	0.9600
C6—C5	1.382 (3)	C17—H17A	0.9600
C7—H7	0.9800	C17—H17B	0.9600
C8—H8	0.9800	C17—H17C	0.9600
C8—C7	1.529 (3)		
			
O1—N1—C2	116.3 (3)	C8—C9—H9C	109.5
O2—N1—O1	125.6 (2)	C8—C10—H10A	109.5
O2—N1—C2	118.1 (2)	C8—C10—H10B	109.5
O3—N2—O4	125.4 (2)	C8—C10—H10C	109.5
O3—N2—C3	117.5 (2)	C9—C8—H8	107.3
O4—N2—C3	117.1 (2)	C9—C8—C7	113.8 (2)
O5—C4—C3	120.13 (19)	C10—C8—H8	107.3
O5—C4—C5	122.0 (2)	C10—C8—C7	110.0 (2)
O5—C16—H16A	109.5	C10—C8—C9	110.8 (2)
O5—C16—H16B	109.5	C11—O7—C7	115.47 (17)
O5—C16—H16C	109.5	C11—N3—C12	122.75 (19)
O6—C5—C4	114.47 (19)	C11—N3—H3	114.4 (19)
O6—C5—C6	125.36 (19)	C12—N3—H3	122.3 (19)
O6—C17—H17A	109.5	C12—C13—H13A	109.3
O6—C17—H17B	109.5	C12—C13—H13B	109.3
O6—C17—H17C	109.5	C12—C13—C14	111.8 (2)
O7—C7—C1	108.12 (17)	C13—C12—H12A	108.8
O7—C7—C8	108.20 (18)	C13—C12—H12B	108.8
O7—C7—H7	108.7	C13—C14—H14A	109.0
O8—C11—O7	123.68 (19)	C13—C14—H14B	109.0
O8—C11—N3	126.3 (2)	C14—C13—H13A	109.3
N3—C12—H12A	108.8	C14—C13—H13B	109.3
N3—C12—H12B	108.8	C14—C15—H15A	109.5
N3—C12—C13	113.99 (19)	C14—C15—H15B	109.5
N3—C11—O7	110.05 (18)	C14—C15—H15C	109.5
C1—C6—H6	119.0	C15—C14—C13	112.9 (2)
C1—C2—N1	120.0 (2)	C15—C14—H14A	109.0
C1—C2—C3	121.2 (2)	C15—C14—H14B	109.0
C1—C7—C8	114.22 (18)	H9A—C9—H9B	109.5
C1—C7—H7	108.7	H9A—C9—H9C	109.5
C2—C3—N2	119.8 (2)	H9B—C9—H9C	109.5
C2—C1—C6	117.2 (2)	H10A—C10—H10B	109.5
C2—C1—C7	122.5 (2)	H10A—C10—H10C	109.5
C3—C4—C5	117.5 (2)	H10B—C10—H10C	109.5
C3—C2—N1	118.75 (19)	H12A—C12—H12B	107.7
C4—O5—C16	115.31 (19)	H13A—C13—H13B	107.9
C4—C3—N2	118.2 (2)	H14A—C14—H14B	107.8
C4—C3—C2	121.9 (2)	H15A—C15—H15B	109.5
C5—O6—C17	117.76 (18)	H15A—C15—H15C	109.5
C5—C6—C1	121.93 (19)	H15B—C15—H15C	109.5
C5—C6—H6	119.0	H16A—C16—H16B	109.5
C6—C1—C7	120.26 (19)	H16A—C16—H16C	109.5
C6—C5—C4	120.2 (2)	H16B—C16—H16C	109.5
C7—C8—H8	107.3	H17A—C17—H17B	109.5
C8—C7—H7	108.7	H17A—C17—H17C	109.5
C8—C9—H9A	109.5	H17B—C17—H17C	109.5
C8—C9—H9B	109.5		
			
O1—N1—C2—C1	−122.5 (2)	C5—C4—C3—C2	4.2 (3)
O1—N1—C2—C3	53.9 (3)	C6—C1—C2—N1	174.4 (2)
O2—N1—C2—C1	56.9 (3)	C6—C1—C2—C3	−1.9 (3)
O2—N1—C2—C3	−126.7 (2)	C6—C1—C7—O7	−36.3 (3)
O3—N2—C3—C4	56.7 (3)	C6—C1—C7—C8	84.2 (2)
O3—N2—C3—C2	−125.9 (3)	C7—O7—C11—O8	−15.0 (3)
O4—N2—C3—C4	−123.5 (2)	C7—O7—C11—N3	165.85 (18)
O4—N2—C3—C2	53.9 (3)	C7—C1—C6—C5	−177.8 (2)
O5—C4—C3—N2	8.2 (3)	C7—C1—C2—N1	−4.3 (3)
O5—C4—C3—C2	−169.1 (2)	C7—C1—C2—C3	179.4 (2)
O5—C4—C5—O6	−8.0 (3)	C9—C8—C7—O7	58.3 (3)
O5—C4—C5—C6	170.6 (2)	C9—C8—C7—C1	−62.1 (3)
N2—C3—C2—N1	4.4 (3)	C10—C8—C7—O7	−66.6 (2)
N2—C3—C2—C1	−179.3 (2)	C10—C8—C7—C1	172.9 (2)
N3—C12—C13—C14	−178.41 (19)	C11—O7—C7—C1	−86.6 (2)
C1—C6—C5—O6	177.1 (2)	C11—O7—C7—C8	149.19 (18)
C1—C6—C5—C4	−1.4 (3)	C11—N3—C12—C13	−106.4 (2)
C2—C1—C7—O7	142.3 (2)	C12—N3—C11—O7	176.03 (19)
C2—C1—C7—C8	−97.2 (3)	C12—N3—C11—O8	−3.1 (4)
C2—C1—C6—C5	3.6 (3)	C12—C13—C14—C15	−178.9 (2)
C3—C4—C5—O6	178.9 (2)	C16—O5—C4—C3	−110.7 (2)
C3—C4—C5—C6	−2.5 (3)	C16—O5—C4—C5	76.3 (3)
C4—C3—C2—N1	−178.3 (2)	C17—O6—C5—C4	172.5 (2)
C4—C3—C2—C1	−2.0 (3)	C17—O6—C5—C6	−6.0 (3)
C5—C4—C3—N2	−178.5 (2)		

### Hydrogen-bond geometry (Å, º)

**Table:**

*D*—H···*A*	*D*—H	H···*A*	*D*···*A*	*D*—H···*A*
N3—H3···O8^i^	0.78 (3)	2.26 (3)	3.042 (3)	176 (3)

Symmetry code: (i) *x*+1/2, −*y*+1, *z*.
